# Fear of Cancer Recurrence in Adult Survivors of Childhood Cancer

**DOI:** 10.1001/jamanetworkopen.2024.36144

**Published:** 2024-10-03

**Authors:** Alex Pizzo, Wendy M. Leisenring, Kayla L. Stratton, Élisabeth Lamoureux, Jessica S. Flynn, Kevin Alschuler, Kevin R. Krull, Lindsay A. Jibb, Paul C. Nathan, Jeffrey E. Olgin, Jennifer N. Stinson, Gregory T. Armstrong, Nicole M. Alberts

**Affiliations:** 1 Department of Psychology, Concordia University, Montréal, Québec, Canada; 2 Clinical Research Division, Fred Hutchinson Cancer Center, Seattle, Washington; 3 Department of Psychology and Biobehavioral Sciences, St Jude Children’s Research Hospital, Memphis, Tennessee; 4 Department of Rehabilitation Medicine, University of Washington, Seattle; 5 The Hospital for Sick Children, Toronto, Ontario, Canada; 6 Department of Medicine, University of California, San Francisco; 7 Department of Epidemiology and Cancer Control, St Jude Children’s Research Hospital, Memphis, Tennessee

## Abstract

**Importance:**

Fear of cancer recurrence is common among survivors of adult-onset cancer and associated with increased distress, functional impairment, and health care utilization. However, little is known about the prevalence and risk factors of fear of cancer recurrence among adult survivors of childhood cancer who are also at high risk for subsequent malignant neoplasms.

**Objective:**

To characterize the prevalence of and risk factors for clinically significant fear of cancer recurrence in adult survivors of childhood cancer.

**Design, Setting, and Participants:**

This cross-sectional investigation included participants recruited from the Childhood Cancer Survivor Study, a retrospective cohort study of long-term childhood cancer survivors treated at 31 institutions between 1970 and 1999 across North America. Participants were recruited and completed psychosocial measures via online survey between October 2018 and April 2019. Cancer and treatment-related variables were abstracted from medical records. Data were analyzed from May 2023 to July 2024.

**Main Outcomes and Measures:**

Clinically significant fear of cancer recurrence was assessed via the Fear of Cancer Recurrence Inventory-Short Form. Poisson regression models estimated prevalence ratios (PRs) with 95% CIs adjusted for age and sex to examine the associations of demographic, disease, treatment, and psychosocial variables with fear of cancer recurrence.

**Results:**

The final sample included 229 adult survivors of childhood cancer (115 female [50.2%]; mean [SD] age, 39.6 [9.9] years; mean [SD] time since diagnosis, 31.7 [8.4] years). Among survivors, 38 (16.6%; 95% CI, 11.6%-21.6%) reported clinically significant fear of cancer recurrence, and an additional 36 (15.7%) reported high fear of cancer recurrence. Clinically significant fear of cancer recurrence was associated with unemployment (PR, 2.5; 95% CI, 1.3-4.8), presence of neurologic chronic health conditions (PR, 3.3; 95% CI, 1.8-6.1), treatment with pelvic radiation (PR, 2.9; 95% CI, 1.5-5.6), and amputation or limb sparing surgery (PR, 2.4; 95% CI, 1.2-4.9). Higher risk of clinically significant fear of cancer recurrence was also associated with having either elevated anxiety or depression (PR, 2.6; 95% CI, 1.2-5.9), having both elevated (PR, 3.2; 95% CI, 1.2-8.4), and perceived poor health status (PR, 3.0; 95% CI, 3.1-9.7).

**Conclusions and Relevance:**

Decades following treatment, one-third of childhood cancer survivors in this study reported elevated fear their cancer will recur or a subsequent malignant neoplasm will develop. Findings suggest that fear of cancer recurrence should be routinely screened, and clinically significant symptoms intervened upon as a part of survivorship care.

## Key Points

**Question:**

What is the prevalence of and related factors for clinically significant fear of cancer recurrence in adult survivors of childhood cancer?

**Findings:**

In this cross-sectional study of 229 North American adults who survived childhood cancer, one-third of survivors reported experiencing elevated fear that their primary cancer will recur or a subsequent malignant neoplasm will develop. Fear of cancer recurrence was associated with chronic health conditions, treatment-related factors, anxiety, depression, and perceived health status.

**Meaning:**

These findings suggest that fear of cancer recurrence is common among adult survivors of childhood cancer and targeted interventions are needed.

## Teaser

This cross-sectional study of adult survivors of childhood cancer examines reported fear of cancer recurrence and associations with chronic health conditions, mental health, and perceived health status.

## Introduction

Fear of cancer recurrence (FCR) is defined as “fear, worry, or concern relating to the possibility that a primary cancer or subsequent malignancy will return or progress to other parts of the body”^1^ and is prevalent among survivors of adult-onset cancer.^2,3,4^ Some vigilance toward pain and associated anxiety is common and likely adaptive in survivors given their increased risk of recurrence of primary cancer and subsequent malignant neoplasms (SMNs).^5,6,7,8,9,10,11,12,13,14^ However, clinically significant levels of FCR (CS-FCR; ie, causing functional impairment and/or distress) are associated with several negative outcomes in adult-onset cancer survivors, including anxiety and depression, reduced quality of life, and overuse of outpatient hospital services.^4,15,16,17^ Common FCR risk factors in this population include younger current age, physical symptoms (eg, fatigue, pain), female sex, metastatic diagnosis, and treatment factors (eg, chemotherapy).^4,6,18,19,20,21,22,23,24,25,26^ Although FCR has been well characterized among adult-onset cancer survivors, less attention has been paid to FCR among adult survivors of childhood cancer, who are at low risk for recurrence of their primary childhood cancer but at high risk for SMNs(approximately 6-fold increased risk compared with the general population).^13,14,27,28,29,30,31,32,33,34,35^

Initial studies examining FCR in childhood cancer survivors show results similar to those in adult-onset cancer.^27,28,29,30,31,32,33,34,35^ However, significant methodological and conceptual limitations exist.^27,28,29,30,31,33,35^ For example, FCR has primarily been examined using unvalidated measures that rely on 1 or 2 items and lack clinical cut-off scores, limiting the ability to fully characterize FCR. The role of chronic pain (ie, pain lasting 3 months or more) has also been overlooked despite the inclusion of acute pain in FCR theoretical models, whereby pain acts as a cancer-related cue, triggering FCR-related thoughts and subsequent anxiety.^36,37,38^ Thus, repeated daily exposure to pain over a prolonged period (ie, chronic pain) may increase the occurrence of FCR. Recent models of FCR also theorize that intolerance of uncertainty,^38^ a personality-based factor associated with anxiety and depression,^39,40,41,42,43,44,45^ may play a role in the appraisal of cancer-related cues as individuals with high levels of intolerance of uncertainty are more likely to interpret ambiguous cues as threatening.^46,47,48^ Despite this, the potential mediating role of intolerance of uncertainty in the childhood cancer context is unclear.

This study aimed to characterize the prevalence of and risk factors for CS-FCR in adult survivors of childhood cancer. Associations between FCR and chronic pain as well as intolerance of uncertainty were examined, followed by an evaluation of the potential mediating role of intolerance of uncertainty in associations between FCR and anxiety and depression. We hypothesized that intolerance of uncertainty would mediate the association between anxiety and FCR as well as depression and FCR.

## Methods

### Participants

A random sample of 700 childhood cancer survivors (ie, diagnosed younger than 21 years of age; surviving 5 years or more) enrolled in the Childhood Cancer Survivor Study (CCSS) was recruited for an ancillary study: Exploring Aspects of Survivors Pain (EASE). CCSS is a retrospective cohort study of long-term childhood cancer survivors treated at 31 institutions between 1970 and 1999 across North America.^49^ Survivors were recruited to EASE via letters, emails, and phone calls and invited to download the Eureka Research app, where all measures were completed. Inclusion criteria for survivors included being older than age 18 years, able to read and speak English, owning a smartphone, and having access to the internet. Participants were recruited and completed measures via the study app between October 2018 and April 2019. Study recruitment and app support were based at St Jude Children’s Research Hospital in Memphis, Tennessee.

Ethics approval was received from St. Jude Children’s Research Hospital and Concordia University for secondary data analysis. Written informed consent was obtained for the original ancillary EASE study. This study followed the Strengthening the Reporting of Observational Studies in Epidemiology (STROBE) reporting guidelines for observational studies.

### Outcomes and Measures

#### Demographic, Diagnostic, and Treatment Factors

Demographic variables were ascertained from medical records and/or self-reported on CCSS surveys and included sex, race and ethnicity, age, marital status, annual income, employment status, and educational attainment (eAppendix 1 in Supplement 1). Self-reported categories for race and ethnicity included American Indian, Black, Chinese, Hispanic, and White; data on race and ethnicity were included as a study variable because previous studies have shown associations between race, ethnicity, and mental health outcomes in medical populations. Cancer-related variables included diagnosis, age at diagnosis, cancer recurrence, and SMNs. Treatment variables were abstracted from medical records and included chemotherapy, radiation, and amputation or limb-sparing surgery. Chronic health conditions were self-reported on surveys, categorized, and graded 1 (asymptomatic or mild) to 4 (life-threatening) based on the National Cancer Institute’s Common Terminology Criteria for Adverse Events.^50^ Only conditions with grades 2 to 4 were included.

#### Fear of Cancer Recurrence

FCR was assessed via the 9-item Fear of Cancer Recurrence Inventory–Short Form (FCRI-SF), which defines *recurrence* as the possibility that cancer could return to the same place or another part of the body (eTable 1 in Supplement 1).^51,52^ Items are rated on a 5-point Likert scale ranging from 0 (not at all) to 4 (a great deal), with higher scores indicating greater FCR.^51,52^ Here a total score below 16 indicates minimal levels, 16 to 21 indicates high levels, and 22 or greater indicates CS-FCR.^52^ See eAppendix 2 in Supplement 1 for additional information regarding the psychometric properties of the FCRI-SF and other measures.

#### Chronic Pain

Chronic pain was assessed via 2 items (ie, “Do you have any persistent or recurrent pain, more than aches and pains that are fleeting?” and “How long have you been experiencing pain?”).^53,54^ Survivors with pain for 3 months or more were categorized as having chronic pain.

#### Depressive Symptoms

Depressive symptoms were assessed via the Patient Health Questionnaire 8-item (PHQ-8).^55,56,57^ Items are rated on a 4-point Likert scale ranging from 0 (not at all) to 3 (nearly every day). Higher scores indicate greater symptom severity, and a total score of 10 or higher is the cut-point for elevated levels of depression.^55,56,57^

#### Anxiety Symptoms

Symptoms of anxiety were assessed via the Generalized Anxiety Disorder 7-item (GAD-7).^58^ Items are rated on a 4-point Likert scale ranging from 0 (not at all) to 3 (nearly every day). Higher scores represent greater symptom severity, and a total score of 10 or higher is the cut-point for elevated levels of anxiety.^58^

#### Self-Perceived Health

Self-perceived health was assessed using one item from the 36-item Short Form Health Survey.^59^ Participants were asked to rate their health on a 5-point Likert scale: 1 (poor), 2 (fair), 3 (good), 4 (very), or 5 (excellent). For analyses, a dichotomous variable defined as poor or fair vs good, very, or excellent was used.

#### Intolerance of Uncertainty Scale-12

The Intolerance of Uncertainty Scale 12-item (IUS-12) was used to measure intolerance of uncertainty.^60,61^ Items are rated on a 5-point Likert scale, with higher scores indicating greater intolerance of uncertainty. Continuous scores on the IUS-12 were used.

#### PROMIS Sleep Disturbance–Short Form

Sleep disturbances were assessed via the National Institute of Health Patient Reported Outcomes Measurement Information System–Sleep Disturbance (PROMIS-SD)–Short Form 8-item. Items are rated on a 5-point Likert scale, with higher scores indicating more sleep difficulties.^62,63^ Continuous scores on the PROMIS-SD were used.

### Statistical Analysis

The percentage of survivors scoring 22 or higher on the FCRI-SF and its corresponding 95% CI was calculated to determine the prevalence of CS-FCR. Comparisons of characteristics between participants and nonparticipants were based on χ^2^ or Fisher exact test to assess potential bias. Univariate modified Poisson regression models with robust standard errors were used to investigate the association between CS-FCR and potential risk factors and estimate prevalence ratios (PR) with 95% CIs.^64^ Before developing multivariable models, we examined the association between FCR and demographic variables (eg, age at EASE survey, sex) selected a priori, which have been shown to be significantly associated with FCR in previous research, to form our base model. Guided by this model, we adjusted for age at EASE survey completion and sex in multivariable models. Separate multivariable regression analyses were conducted examining associations between CS-FCR and (1) demographic variables, (2) cancer diagnosis, (3) chronic health conditions, (4) treatment exposures, and (5) psychosocial factors. Factors included in the final models were selected stepwise using the Akaike information criterion as an aid to determine the most parsimonious model.

Regarding the mediation model, we followed the recommendations summarized in Hayes.^65^ Mediation was defined as occurring if the indirect effect was significantly different from zero. To calculate the indirect effect, we multiplied the effect of elevated anxiety and depression on intolerance of uncertainty (*a_1_* and *a_2_*) by the effect of intolerance of uncertainty on CS-FCR (effect *b*). To meaningfully interpret any mediation effects, we estimated indirect effects (*a_1_ × b* and *a_2_ × b*) with 1000 bootstrap samples, reporting confidence intervals based on the 2.5 and 97.5 percentile of the distribution and testing significance of the estimate utilizing the standard deviation of the distribution. Mediation analyses were adjusted for age at EASE survey completion and sex.

Missing data are summarized (Table 1). If a variable with missing data was used in a particular analysis, only survivors with complete data were used. All assumptions required for statistical analyses were met. Nominal 2-sided *P* values are shown with a significance level set at *P* < .05. Within each multivariable regression model, adjusted *P* values accounting for overall false discovery rate (FDR) are also shown.^66^ Analyses were conducted using SAS statistical software, version 9.4 (SAS Institute).

**Table 1.** Demographics and Clinical Characteristics of Survivors of Childhood Cancer

Characteristics	Participants, No. (%)^a^		
	Overall (n = 229)	Nonclinically significant fear of cancer recurrence (n = 191)	Clinically significant fear of cancer recurrence (n = 38)
Age at study, mean (SD), y	39.6 (9.9)	39.6 (10.3)	39.5 (7.8)
Age at diagnosis, mean (SD), y	7.9 (6.1)	7.8 (6.0)	8.4 (6.2)
Time since diagnosis, mean (SD), y	31.7 (8.4)	31.8 (8.4)	31.0 (8.2)
Sex			
Male	114 (49.8)	100 (52.4)	14 (36.8)
Female	115 (50.2)	91 (47.6)	24 (63.2)
Race and ethnicity			
American Indian	1 (0.4)	1 (0.5)	0
Black	6 (2.6)	5 (2.6)	1 (2.6)
Chinese	1 (0.4)	1 (0.5)	0 (0.0)
Hispanic^b^	11 (4.8)	9 (4.7)	2 (5.3)
White, Non-Hispanic	205 (89.5)	170 (89.0)	35 (92.1)
White, Hispanic ethnicity unknown	5 (2.2)	5 (2.6)	0
Education			
Completed high school	29 (12.7)	21 (11.0)	8 (21.1)
Some college or college graduate	148 (64.6)	120 (62.8)	28 (73.7)
Postgraduate	52 (22.7)	50 (26.2)	2 (5.3)
Employment			
Full-time	139 (66.5)	121 (69.1)	18 (52.9)
Part-time	30 (14.4)	27 (15.4)	3 (8.8)
Not employed	40 (19.1)	27 (15.4)	13 (38.2)
Unknown	20	16	4
Diagnosis			
Leukemia	79 (34.5)	68 (35.6)	11 (28.9)
CNS tumor	24 (10.5)	18 (9.4)	6 (15.8)
Lymphomas (HL, NHL)	47 (20.5)	41 (21.5)	6 (15.8)
Wilms, neuroblastoma, STS	51 (22.3)	43 (22.5)	8 (21.1)
Bone cancer	28 (12.2)	21 (11.0)	7 (18.4)
Married			
Yes	129 (63.2)	109 (63.4)	20 (62.5)
No	75 (36.8)	63 (36.6)	12 (37.5)
Unknown	25	19	6
Location			
Metropolitan (RUCA 1-3)	177 (79.7)	152 (81.3)	25 (71.4)
Nonmetropolitan (RUCA 4-10)	45 (20.3)	35 (18.7)	10 (28.6)
Unknown	7	4	3
Endocrine condition (grade 2-4)			
Yes	80 (34.9)	64 (33.5)	16 (42.1)
No	149 (65.1)	127 (66.5)	22 (57.9)
Respiratory condition (grade 2-4)			
Yes	22 (9.6)	17 (8.9)	5 (13.2)
No	207 (90.4)	174 (91.1)	33 (86.8)
Cardiovascular condition (grade 2-4)			
Yes	77 (33.6)	63 (33.0)	14 (36.8)
No	152 (66.4)	128 (67.0)	24 (63.2)
GI condition (grade 2-4)			
Yes	27 (11.8)	22 (11.5)	5 (13.2)
No	202 (88.2)	169 (88.5)	33 (86.8)
Musculoskeletal condition (grade 2-4)			
Yes	20 (8.7)	17 (8.9)	3 (7.9)
No	209 (91.3)	174 (91.1)	35 (92.1)
Neurological condition (grade 2-4)			
Yes	34 (14.8)	19 (9.9)	15 (39.5)
No	195 (85.2)	172 (90.1)	23 (60.5)
Physical health status			
Poor, fair	34 (15.1)	18 (9.5)	16 (44.4)
Good, very good, excellent	191 (84.9)	171 (90.5)	20 (55.6)
Unknown	4	2	2
Chemotherapy			
Yes	186 (85.3)	156 (85.7)	30 (83.3)
No	32 (14.7)	26 (14.3)	6 (16.7)
Unknown	12	10	2
Vinca alkaloids			
Yes	162 (74.3)	138 (75.8)	24 (66.7)
No	56 (25.7)	44 (24.2)	12 (33.3)
Unknown	11	9	2
Platinum			
Yes	26 (11.9)	20 (11.0)	6 (16.7)
No	192 (88.1)	162 (89.0)	30 (83.3)
Unknown	11	9	2
Intravenous methotrexate ≥10 000 mg/m^2^			
Yes	25 (11.7)	22 (12.2)	3 (8.8)
No	189 (88.3)	158 (87.8)	31 (91.2)
Unknown	15	11	4
Intrathecal methotrexate			
Yes	89 (41.0)	76 (41.8)	13 (37.1)
No	128 (59.0)	106 (58.2)	22 (62.9)
Unknown	12	9	3
Radiation			
Yes	98 (44.7)	82 (44.8)	16 (44.4)
No	121 (55.3)	101 (55.2)	20 (55.6)
Unknown	10	8	2
Cranial radiation			
Yes	46 (21.1)	39 (21.4)	7 (19.4)
No	172 (78.9)	143 (78.6)	29 (80.6)
Other radiation to head			
Yes	10 (4.6)	9 (4.9)	1 (2.8)
No	208 (95.4)	173 (95.1)	35 (97.2)
Neck radiation			
Yes	32 (14.7)	26 (14.3)	6 (16.7)
No	186 (85.3)	156 (85.7)	30 (83.3)
Chest radiation			
Yes	34 (15.6)	28 (15.4)	6 (16.7)
No	184 (84.4)	154 (84.6)	30 (83.3)
Abdomen radiation			
Yes	35 (16.1)	27 (14.8)	8 (22.2)
No	183 (83.9)	155 (85.2)	28 (77.8)
Pelvis radiation			
Yes	25 (11.5)	16 (8.8)	9 (25.0)
No	193 (88.5)	166 (91.2)	27 (75.0)
Limb radiation			
Yes	8 (3.7)	6 (3.3)	2 (5.6)
No	210 (96.3)	176 (96.7)	34 (94.4)
Nonbrain radiation			
Yes	63 (28.9)	50 (27.5)	13 (36.1)
No	155 (71.1)	132 (72.5)	23 (63.9)
Surgery			
Yes	162 (70.7)	136 (71.2)	26 (68.4)
No	67 (29.3)	55 (28.8)	12 (31.6)
Amputation			
Yes	12 (5.2)	11 (5.8)	1 (2.6)
No	217 (94.8)	180 (94.2)	37 (97.4)
Limb sparing			
Yes	11 (4.8)	5 (2.6)	6 (15.8)
No	218 (95.2)	186 (97.4)	32 (84.2)
Amputation or limb sparing			
Yes	23 (10.0)	16 (8.4)	7 (18.4)
No	206 (90.0)	175 (91.6)	31 (81.6)
Any recurrence			
Yes	21 (9.2)	16 (8.4)	5 (13.2)
No	208 (90.8)	175 91.6	33 (86.8)
Any SMN (non-NMSC)			
Yes	17 (7.4)	15 (7.9)	2 (5.3)
No	212 (92.6)	176 (92.1)	36 (94.7)

Abbreviations: CNS, central nervous system; FCR, fear of cancer recurrence; GI, gastrointestinal; HL, Hodgkin lymphoma; NHL, non-Hodgkin lymphoma; NMSC, nonmelanoma skin cancer; RUCA, rural-urban commuting area codes; SMN, second malignant neoplasms; STS, sarcomas, soft tissue.

^a^ Percentages among those with known values; 11 survivors (2 with FCR, 9 with no FCR) were missing information on radiation location.

^b^ Hispanic ethnicity included 3 participants identifying as Mexican, Mexican Hispanic, Mexican American, or Chicano; 1 as Cuban; 5 as Latino or Spanish Origin; and 2 as type unknown.

## Results

Of 700 invited CCSS survivors, 229 (32.7%) consented and completed study procedures, with a mean (SD) age at diagnosis and study completion of 7.9 (6.1) years and 39.6 (9.9) years, respectively (Table 1; eFigure 1 in Supplement 1). The most common diagnosis was leukemia (79 [34.5%]). Of survivors in our sample, 21 (9.2%) experienced a recurrence of their primary cancer and 17 (7.4%) had been diagnosed with a SMN. Ninety-four survivors (41.0%) reported experiencing chronic pain. Demographic and other descriptive data compared EASE participants and nonparticipating members of the invited CCSS cohort, finding significant differences in marital status, race and ethnicity, and chronic endocrine conditions (eTable 2 in Supplement 1).

### Prevalence of FCR

The mean (SD) score on the FCRI-SF was 12.0 (9.1) (range, 0-36.0) (Table 2). Thirty-eight participants (16.6%; 95% CI, 11.8-21.4) reported experiencing CS-FCR. A further 36 (15.7%) and 155 (67.7%) participants reported experiencing high and minimal levels of FCR, respectively. Among survivors with chronic pain, 24 (25.5%) and 13 (13.8%) reported experiencing clinical and high levels of FCR, respectively.

**Table 2.** Psychosocial Characteristics of Survivors With and Without Clinically Significant Fear of Cancer Recurrence

Characteristic	Overall, No. (%) (n = 229)	Without clinical fear of cancer recurrence, No. (%) (n = 191)	With clinical fear of cancer recurrence, No. (%) (n = 38)
FCRI-SF, mean (SD)^a^	12.0 (9.1) [0-36.0]	9.5 (6.9) [0-21.0]	25.7 (3.6) [22.0-36.0]
PHQ-8, mean (SD)^b^			
Mean (SD) [range]	6.3 (6.4) [0-24.0]	5.2 (5.6) [0-23.0]	11.9 (6.9) [0-24.0]
Mild	170 (74.2)	153 (80.1)	17 (44.7)
Elevated	59 (25.8)	38 (19.9)	21 (55.3)
GAD-7^c^			
Mean (SD) [range]	5.8 (6.2) [0-21.0]	4.7 (5.4) [0.0-21.0]	11.9 (6.2) [0-21.0]
Mild	174 (76.0)	158 (82.7)	16 (42.1)
Elevated	55 (24.0)	33 (17.3)	22 (57.9)
PHQ-8 and GAD-7^d^			
Depression only	23 (10.0)	18 (9.4)	5 (13.2)
Anxiety only	19 (8.3)	13 (6.8)	6 (15.8)
Depression and anxiety	36 (15.7)	20 (10.5)	16 (42.1)
PROMIS-SD, mean (SD)^e^	52.3 (3.3) [41.1-61.7]	52.1 (3.3) [43.8-61.7]	52.9 (3.2) [41.1-57.9]
IUS-12, mean (SD)^f^	25.9 (10.7) [12.0-59.0]	24.4 (9.5) [12.0-59.0]	34.2 (12.0) [14.0-58.0]
Chronic pain	94 (41.0)	70 (36.6)	24 (63.2)
Perceived health, mean (SD)^g^	2.4 (1.1) [1.0-5.0]	2.3 (1.0) [1.0-5.0]	3.3 (1.0) [1.0-5.0]

Abbreviations: CS-FCR, clinically significant fear of cancer recurrence; FCRI-SF, Fear of Cancer Recurrence Inventory–Short Form; GAD-7, Generalized Anxiety Disorder-7; IUS-12, Intolerance of Uncertainty Scale-12; PHQ-8, Patient Health Questionnaire-8; PROMIS-SD, Patient Reported Outcomes Measurement Information System–Sleep Disturbance.

^a^ Depression only described survivors who only had elevated levels of depression based on the PHQ-8; anxiety only, survivors who only had elevated levels of anxiety based on the GAD-7; depression and anxiety, survivors who had elevated levels of both anxiety and depression based on the PHQ-8 and GAD-7.

^b^ Nine-item 5-point Likert scale (0-4 range); total scale of 36 points, with scores <16 indicating minimal levels, 16-21 indicating high levels, and ≥22 or greater indicating CS-FCR.

^c^ Four-point Likert scale (0-3); total scores ≥10 indicate elevated levels of depression.

^d^ Four-point Likert scale (0-3); total scores ≥10 indicate elevated levels of anxiety.

^e^ Five-point Likert scale (1-5), with higher scores indicating increasing sleep difficulties.

^f^ Five-point Likert scale (1-5), with higher scores indicating greater intolerance of uncertainty.

^g^ Assessed with 1 item from the 36-item Short Form Health Survey using a 5-point Likert scale (1 [poor] to 5 [excellent]).

### Risk Factors Associated With Clinically Significant FCR

Initial univariate models assessed the effect of individual risk factors on CS-FCR (eTable 3 in Supplement 1). In our base model, female participants (PR, 2.0; 95% CI 1.03-3.7) were more likely to experience CS-FCR than male participants, although this result was not significant when FDR-adjusted *P* values were used (Table 3, Model 0). In a final multivariable model with demographic variables, survivors who were unemployed (PR, 2.5; 95% CI, 1.3-4.8) or who completed some college or an undergraduate degree (PR, 4.9; 95% CI 1.2-19.4) were more likely to experience CS-FCR (Table 3, Model 1). No associations were observed between CS-FCR and recurrence of the primary cancer or development of a SMN (eTable 3 in Supplement 1).

**Table 3.** Multivariate Models Assessing Risk Factors for Clinically Significant Fear of Cancer Recurrence

Risk factor^a^	Prevalence ratio (95% CI)	*P* value	Adjusted *P* value^b^
Model 0 (base model)			
Age at cancer diagnosis (per 10 y)	0.7 (0.4-1.4)	.34	.62
Age at EASE survey completion (per 10 y)	1.2 (0.7-2.1)	.46	.62
Sex			
Male	1 [Reference]	[Reference]	[Reference]
Female	2.0 (1.03-3.7)	.04	.16
Race			
White, non-Hispanic	1 [Reference]	[Reference]	[Reference]
Other race or ethnicity^c^	0.8 (0.2-2.3)	.62	.62
Model 1 (demographic factors)			
Employment			
Employed part or full time	1 [Reference]	[Reference]	[Reference]
Not employed	2.5 (1.3-4.8)	.004	.01
Education			
Postgraduate	1 [Reference]	[Reference]	[Reference]
Completed high school	3.0 (0.5-15.9)	.21	.21
Some college/college graduate	4.9 (1.2-19.4)	.02	.04
Model 2 (diagnosis)			
Leukemia	1 [Reference]	[Reference]	[Reference]
CNS	2.1 (0.8-5.5)	.13	.28
Lymphomas (HL, NHL)	1.1 (0.4-3.0)	.83	.28
Bone cancer	2.0 (0.8-4.8)	.14	.83
Wilms, neuroblastoma, soft tissue sarcoma	1.2 (0.5-3.2)	.65	.83
Model 3 (chronic health conditions)			
Neurological conditions			
No	1 [Reference]	[Reference]	[Reference]
Yes	3.3 (1.8-6.1)	<.001	<.001
Model 4 (treatment exposures)			
Amputation or limb sparing			
No	1 [Reference]	[Reference]	[Reference]
Yes	2.4 (1.2-4.9)	.01	.01
Pelvic radiation			
No	1 [Reference]	[Reference]	[Reference]
Yes	2.9 (1.5-5.6)	.002	.004
Model 5 (psychosocial factors)			
Depression and anxiety			
Neither elevated	1 [Reference]	[Reference]	[Reference]
Either elevated	2.6 (1.2-5.9)	.02	.04
Both elevated	3.2 (1.2-8.4)	.02	.04
Health related quality of life			
Excellent, very good, good	1 [Reference]	[Reference]	[Reference]
Fair, poor	3.0 (1.6-5.9)	<.001	<.001
Chronic pain			
No	1 [Reference]	[Reference]	[Reference]
Yes	1.2 (0.6-2.4)	.64	.64
Intolerance of uncertainty (per 5-unit increment)	1.1 (0.9-1.3)	.48	.60

Abbreviations: CNS, central nervous system; HL, Hodgkin lymphoma; NHL, non-Hodgkin lymphoma.

^a^ Represents adjustment for multiple comparisons, using method of Benjamini and Hochberg.

^b^ Models 1-5 adjusted for age at EASE (Exploring Aspects of Survivors Pain) survey completion and sex.

^c^ Including American Indian, Black, Chinese, and Hispanic.

Survivors with a neurological condition (PR, 3.3; 95% CI, 1.8-6.1) were more likely to experience CS-FCR (Table 3, Model 3). Survivors who underwent pelvic radiation (PR, 2.9; 95% CI, 1.5-5.6) or limb-sparing or amputation surgery (PR, 2.4; 95% CI, 1.2-4.9) were at higher risk of CS-FCR (Table 3, Model 4).

Regarding psychosocial factors, survivors with either elevated depression or anxiety (PR, 2.6; 95% CI, 1.2-5.9) or both (PR, 3.2; 95% CI, 1.2-8.4) were more likely to experience CS-FCR (Table 3, Model 5). Survivors who rated their health as poor or fair (PR, 3.0; 95% CI, 1.6-5.9) were also at greater risk of CS-FCR compared with good, very good, or excellent (Table 3, Model 5). Although significant in univariate analyses, chronic pain (PR, 1.2; 95% CI, 0.6-2.4) and intolerance of uncertainty (PR, 1.1; 95% CI, 0.9-1.3) were not associated with CS-FCR in this model.

### Mediation Analyses

Multiple mediation models with indicators representing various combinations of anxiety and depression dichotomized were calculated. Results discussed are from Model C (eTable 4 in Supplement 1). In examining whether intolerance of uncertainty mediated the association between FCR and anxiety, a direct effect of elevated anxiety on CS-FCR, with or without elevated depression, was found (*c_1_’* = 1.37; 95% CI, 0.57-2.17) (Figure). After bootstrapping, a significant indirect effect was also observed (*a_1_ × b* = 0.42; 95% CI, 0.02-0.82). There was no statistically significant direct effect of elevated depression, without anxiety, on CS-FCR (*c_2_’* = 0.98; 95% CI, −0.03 to 2.00; *P* = .06) and the indirect effect (*a_2_ × b* = 0.11; 95% CI −0.08 to 0.29) was not significant. These findings suggest intolerance of uncertainty is a partial mediator of the association between FCR and anxiety.

**Figure.**
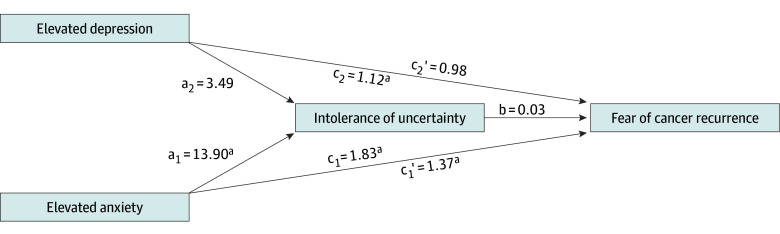
Simple Mediation Model Examining the Mediational Effect of Intolerance of Uncertainty on the Association Between Clinically Significant Fear of Cancer Recurrence and Anxiety and Depression Model adjusted for age at survey completion and sex. Elevated anxiety indicates elevated levels of anxiety with or without depression; elevated depression, for depression only. a_1_ represents effect of elevated anxiety on intolerance of uncertainty; a_2_, effect of elevated depression on intolerance of uncertainty; b, effect of intolerance of uncertainty on clinically significant fear of cancer recurrence; c_1_, effect of elevated anxiety on clinically significant fear of cancer recurrence; c_1_’, direct effect of elevated anxiety on clinically significant cancer recurrence when controlling for intolerance of uncertainty; c_2_, effect of elevated depression on clinically significant cancer recurrence; c_2_’, direct effect of elevated depression on clinically significant fear of cancer recurrence when controlling for intolerance of uncertainty. ^a^*P* < .05.

## Discussion

In this novel study using a validated and comprehensive measure,^51,52^ we identified approximately 1 in 3 adult survivors of childhood cancer experienced FCR at a severity likely to affect their functioning and where psychological intervention would be recommended.^52^ These findings underscore the substantial psychological and functional burden of FCR and suggest health care professionals should routinely assess FCR as a part of providing comprehensive care to long-term survivors. The FCRI-SF and newly developed single-item screeners of FCR are well suited for rapid administration and interpretation within clinical settings.^52,67,68^

Similar to studies in young childhood cancer survivors, no associations emerged between FCR and age at survey completion.^30,34,35^ Unemployment was associated with CS-FCR. As previous studies have shown associations between elevated FCR and functional impairment,^4^ survivors with elevated FCR may be experiencing similar impairment and associated difficulties working.

No associations emerged between cancer type and FCR. Previous research in this area is mixed, with no consistent associations observed across studies.^4,24,28,30^ Contrary to previous findings among childhood cancer survivors,^28,30^ no associations emerged between FCR and recurrence of the primary cancer or a SMN. Given the limited number of survivors with a primary recurrence or a SMN, it is possible the analyses were underpowered to detect a significant effect.

Consistent with our prior work,^30^ survivors with either elevated anxiety or depression or both were at higher risk of CS-FCR. Some chronic neurological conditions are associated with symptoms that could be misinterpreted as a sign of cancer such as pain and weakness.^69,70,71^ Thus, misinterpretation of these types of symptoms may lead to FCR.

Limb-sparing surgery or amputation and pelvic radiation were also associated with increased risk of CS-FCR. The resulting effects of limb-sparing surgery and amputation may activate survivors’ FCR-related thoughts due to more visible and constant reminders of the cancer experience. As pelvic radiation is associated with long-term adverse effects (eg, diarrhea, abdominal pain)^72,73,74^ that can mimic symptoms of bowel and urinary tract cancers,^75,76^ survivors may misinterpret these symptoms as a sign of a new malignant neoplasm.

Consistent with prior research, survivors with either elevated anxiety or depression or both were at higher risk of CS-FCR.^4,27,34,35^ Given the anxiety-based nature of FCR and its high comorbidity with anxiety disorders, as well as between anxiety and depression,^77^ it is not surprising associations between these variables were found. Consistent with previous studies,^4^ survivors who rated their health as poor or fair were more likely to experience CS-FCR. Chronic pain and intolerance of uncertainty were significant predictors in univariate analyses but did not remain significant in the multivariable model. As nearly 40% of survivors with chronic pain also experienced high or greater levels of FCR, these 2 constructs appear to be intricately linked. However, findings from the multivariable model suggest that psychological factors likely play a stronger role in FCR.

Our findings also indicate intolerance of uncertainty is a partial mediator of the association between FCR and anxiety. Consistent with theoretical models of FCR,^38^ intolerance of uncertainty may act as a personality-based factor that colors survivors’ experience of anxiety in relation to FCR. Notably, when our mediational analyses are considered in light of our multivariable psychosocial model, anxiety and depression appear to have a stronger effect on FCR than intolerance of uncertainty. Thus, anxiety and depression may play a more substantial role in the development and maintenance of FCR than intolerance of uncertainty.

Regarding treatment, psychological interventions such as cognitive behavioral therapy and acceptance and commitment therapy, as well as mind-body interventions, are effective in reducing FCR in survivors of adult-onset cancer^78,79,80^; and may also be effective in childhood cancer survivors. Interventions such as cognitive bias modification have also shown promise.^81^ Furthermore, survivors may benefit from less intensive psychological interventions (eg, psychoeducation, peer support groups) to prevent FCR from transitioning to clinical levels.^52^

### Limitations

Study limitations should be noted. As adult survivors are at low risk for recurrence of their primary childhood cancer but at high risk for SMNs, it is unclear to what degree each component is driving the overall prevalence. Studies specifically examining fear around the primary cancer recurring and of SMNs are needed. Nonetheless, that one-third of survivors have elevated or CS-FCR is striking. Although only 32% of contacted survivors participated, minimal differences were observed between participants and nonparticipants in the larger CCSS cohort. Only 1 item was used to assess self-perceived health, limiting the measurement of this variable. A cross-sectional design was used; therefore, inferences regarding causation or directionality cannot be made. Ideally, mediation would be evaluated using longitudinal data to provide stronger evidence of a causal relationship. As previously shown, a cross-sectional mediation analysis could lead to erroneous conclusions.^82^ Therefore, our results showing the effect of anxiety on FCR is mediated in part by intolerance of uncertainty should be considered hypothesis generating and be confirmed in a longitudinal analysis with data collected at 3 waves. Survivors were eligible for the current study if they had internet and smartphone access, which may have biased our sample. Future studies should integrate alternative survey completion methods (eg, paper and pencil). The racial and ethnic diversity of the sample was also limited, and future work with more diverse populations of survivors are needed.

## Conclusions

Despite being decades from the completion of cancer treatments, one-third of adult survivors of childhood cancer experience elevated fear their primary cancer will recur or a SMN will develop. In addition to providing one of the first characterizations of FCR among adult survivors of childhood cancer, the current study demonstrated the robust role of anxiety, depression, and survivors’ perception of their health as risk factors for CS-FCR. Moreover, our findings help lay the groundwork for an improved understanding of FCR among long-term survivors, which is vital to informing psychological screening and intervention efforts.

## Notes

Supplement 1. **eAppendix 1.** Additional Information Regarding Selection of Demographic Variables **eAppendix 2.** Additional Information About Psychometric Properties and Prior Use of Study Measures **eTable 1.** Items on the Fear of Cancer Recurrence Inventory - Short Form **eTable 2.** Demographics and Clinical Characteristics of Participants and Non-Participants **eTable 3.** Univariate Models Assessing Risk Factors for Clinically Significant Fear of Cancer Recurrence **eTable 4.** Models for Intolerance of Uncertainty as a Mediator of the Effect of Elevated Anxiety and/or Depression on Clinical Fear of Cancer Recurrence **eFigure.** Participant Flow Diagram
eReferences

Supplement 2.
Data Sharing Statement

## References

[1] Lebel S, Ozakinci G, Humphris G, . From normal response to clinical problem: definition and clinical features of fear of cancer recurrence. Support Care Cancer. 2016;24(8):3265-3268. doi:10.1007/s00520-016-3272-5 27169703

[2] Luigjes-Huizer YL, Tauber NM, Humphris G, . What is the prevalence of fear of cancer recurrence in cancer survivors and patients? A systematic review and individual participant data meta-analysis. Psychooncology. 2022;31(6):879-892. doi:10.1002/pon.5921 35388525 PMC9321869

[3] Shay LA, Carpentier MY, Vernon SW. Prevalence and correlates of fear of recurrence among adolescent and young adult versus older adult post-treatment cancer survivors. Support Care Cancer. 2016;24(11):4689-4696. doi:10.1007/s00520-016-3317-9 27387913 PMC5033688

[4] Simard S, Thewes B, Humphris G, . Fear of cancer recurrence in adult cancer survivors: a systematic review of quantitative studies. J Cancer Surviv. 2013;7(3):300-322. doi:10.1007/s11764-013-0272-z 23475398

[5] Corrado G, Salutari V, Palluzzi E, Distefano MG, Scambia G, Ferrandina G. Optimizing treatment in recurrent epithelial ovarian cancer. Expert Rev Anticancer Ther. 2017;17(12):1147-1158. doi:10.1080/14737140.2017.1398088 29086618

[6] Koch L, Jansen L, Brenner H, Arndt V. Fear of recurrence and disease progression in long-term (≥5 years) cancer survivors-a systematic review of quantitative studies: fear of recurrence and disease progression in long-term cancer survivors. Psychooncology. 2013;22(1):1-11. doi:10.1002/pon.3022 22232030

[7] Mahvi DA, Liu R, Grinstaff MW, Colson YL, Raut CP. Local cancer recurrence: the realities, challenges, and opportunities for new therapies. CA Cancer J Clin. 2018;68(6):488-505. doi:10.3322/caac.21498 30328620 PMC6239861

[8] Martini N, Bains MS, Burt ME, . Incidence of local recurrence and second primary tumors in resected stage I lung cancer. J Thorac Cardiovasc Surg. 1995;109(1):120-129. doi:10.1016/S0022-5223(95)70427-2 7815787

[9] Mertens AC, Yasui Y, Neglia JP, . Late mortality experience in five-year survivors of childhood and adolescent cancer: the Childhood Cancer Survivor Study. JCO. 2001;19(13):3163-3172. doi:10.1200/JCO.2001.19.13.3163 11432882

[10] Spronk I, Schellevis FG, Burgers JS, De Bock GH, Korevaar JC. Incidence of isolated local breast cancer recurrence and contralateral breast cancer: a systematic review. Breast. 2018;39:70-79. doi:10.1016/j.breast.2018.03.011 29621695

[11] Wasilewski-Masker K, Liu Q, Yasui Y, . Late recurrence in pediatric cancer: a report from the Childhood Cancer Survivor Study. J Natl Cancer Inst. 2009;101(24):1709-1720. doi:10.1093/jnci/djp417 19966206 PMC2800799

[12] Mariotto AB, Rowland JH, Ries LAG, Scoppa S, Feuer EJ. Multiple cancer prevalence: a growing challenge in long-term survivorship. Cancer Epidemiol Biomarkers Prev. 2007;16(3):566-571. doi:10.1158/1055-9965.EPI-06-0782 17372253

[13] Armstrong GT, Liu Q, Yasui Y, . Late mortality among 5-year survivors of childhood cancer: a summary from the Childhood Cancer Survivor Study. J Clin Oncol. 2009;27(14):2328-2338. doi:10.1200/JCO.2008.21.1425 19332714 PMC2677921

[14] Turcotte LM, Neglia JP, Reulen RC, . Risk, risk factors, and surveillance of subsequent malignant neoplasms in survivors of childhood cancer: a review. J Clin Oncol. 2018;36(21):2145-2152. doi:10.1200/JCO.2017.76.7764 29874133 PMC6075849

[15] Deimling GT, Bowman KF, Sterns S, Wagner LJ, Kahana B. Cancer-related health worries and psychological distress among older adult, long-term cancer survivors. Psychooncology. 2006;15(4):306-320. doi:10.1002/pon.955 16041841

[16] Simard S, Savard J, Ivers H. Fear of cancer recurrence: specific profiles and nature of intrusive thoughts. J Cancer Surviv. 2010;4(4):361-371. doi:10.1007/s11764-010-0136-8 20617394

[17] Strollo SE, Fallon EA, Gapstur SM, Smith TG. Cancer-related problems, sleep quality, and sleep disturbance among long-term cancer survivors at 9-years post diagnosis. Sleep Med. 2020;65:177-185. doi:10.1016/j.sleep.2019.10.008 32029206

[18] Brown SL, Fisher P, Hope-Stone L, . Fear of cancer recurrence and adverse cancer treatment outcomes: predicting 2- to 5-year fear of recurrence from post-treatment symptoms and functional problems in uveal melanoma survivors. J Cancer Surviv. 2023;17(1):187-196. doi:10.1007/s11764-021-01129-0 34850324

[19] Cho D, Chu Q, Lu Q. Associations among physical symptoms, fear of cancer recurrence, and emotional well-being among Chinese American breast cancer survivors: a path model. Support Care Cancer. 2018;26(6):1755-1761. doi:10.1007/s00520-017-4010-3 29243170

[20] Crist JV, Grunfeld EA. Factors reported to influence fear of recurrence in cancer patients: a systematic review. Psychooncology. 2013;22(5):978-986. doi:10.1002/pon.3114 22674873

[21] Lebel S, Beattie S, Arès I, Bielajew C. Young and worried: age and fear of recurrence in breast cancer survivors. Health Psychol. 2013;32(6):695-705. doi:10.1037/a0030186 23088176

[22] Meissner VH, Olze L, Schiele S, . Fear of cancer recurrence and disease progression in long-term prostate cancer survivors after radical prostatectomy: a longitudinal study. Cancer. 2021;127(22):4287-4295. doi:10.1002/cncr.33836 34358337

[23] McGinty HL, Small BJ, Laronga C, Jacobsen PB. Predictors and patterns of fear of cancer recurrence in breast cancer survivors. Health Psychol. 2016;35(1):1-9. doi:10.1037/hea0000238 26030308

[24] Thewes B, Kaal SEJ, Custers JAE, . Prevalence and correlates of high fear of cancer recurrence in late adolescents and young adults consulting a specialist adolescent and young adult (AYA) cancer service. Support Care Cancer. Published online November 22, 2017. doi:10.1007/s00520-017-3975-2 PMC5876258 29168035

[25] Van De Wal M, Van De Poll-Franse L, Prins J, Gielissen M. Does fear of cancer recurrence differ between cancer types? A study from the population-based PROFILES registry: PROFILES–fear of cancer recurrence in cancer survivors. Psychooncology. 2016;25(7):772-778. doi:10.1002/pon.4002 26464337

[26] Yang Y, Wen Y, Bedi C, Humphris G. The relationship between cancer patient’s fear of recurrence and chemotherapy: a systematic review and meta-analysis. J Psychosom Res. 2017;98:55-63. doi:10.1016/j.jpsychores.2017.05.002 28554373

[27] Cunningham SJ, Patton M, Schulte F, Richardson PA, Heathcote LC. Worry about somatic symptoms as a sign of cancer recurrence: prevalence and associations with fear of recurrence and quality of life in survivors of childhood cancer. Psychooncology. 2021;30(7):1077-1085. doi:10.1002/pon.5647 33544422

[28] Kelada L, Wakefield CE, Heathcote LC, . Perceived cancer-related pain and fatigue, information needs, and fear of cancer recurrence among adult survivors of childhood cancer. Patient Educ Couns. 2019;102(12):2270-2278. doi:10.1016/j.pec.2019.06.022 31257099

[29] Koutná V, Blatný M, Jelínek M. Posttraumatic stress and growth in childhood cancer survivors: considering the pathways for relationship. J Psychosoc Oncol. 2021;39(1):105-117. doi:10.1080/07347332.2020.1789907 32729397

[30] McDonnell GA, Brinkman TM, Wang M, . Prevalence and predictors of cancer-related worry and associations with health behaviors in adult survivors of childhood cancer. Cancer. 2021;127(15):2743-2751. doi:10.1002/cncr.33563 33844273 PMC8323824

[31] Patton M, Racine N, Afzal AR, . The pain of survival: Prevalence, patterns, and predictors of pain in survivors of childhood cancer. Health Psychology. 2021;40(11):784. doi:10.1037/hea0001116 34914483

[32] Tutelman PR, Chambers CT, Noel M, . Pain and fear of cancer recurrence in survivors of childhood cancer. Clin J Pain. 2022;38(7):484-491. doi:10.1097/AJP.0000000000001049 35686578

[33] Tutelman PR, Heathcote LC. Fear of cancer recurrence in childhood cancer survivors: a developmental perspective from infancy to young adulthood. Psychooncology. 2020;29(11):1959-1967. doi:10.1002/pon.5576 33068463

[34] Tutelman PR, Chambers CT, Heathcote LC, . Measuring fear of cancer recurrence in survivors of childhood cancer: development and preliminary validation of the Fear of Cancer Recurrence Inventory (FCRI)-Child and Parent versions. Psychooncology. 2022;31(6):911-919. doi:10.1002/pon.5879 35018689

[35] Wroot H, Afzal AR, Forbes C, . Fear of cancer recurrence among survivors of childhood cancer. Psychooncology. 2020;29(7):1132-1140. doi:10.1002/pon.5387 32281171

[36] Heathcote LC, Eccleston C. Pain and cancer survival: a cognitive-affective model of symptom appraisal and the uncertain threat of disease recurrence. Pain. 2017;158(7):1187-1191. doi:10.1097/j.pain.0000000000000872 28195857

[37] Simonelli LE, Siegel SD, Duffy NM. Fear of cancer recurrence: a theoretical review and its relevance for clinical presentation and management: fear of recurrence. Psychooncology. 2017;26(10):1444-1454. doi:10.1002/pon.4168 27246348

[38] Lebel S, Maheu C, Tomei C, . Towards the validation of a new, blended theoretical model of fear of cancer recurrence. Psychooncology. 2018;27(11):2594-2601. doi:10.1002/pon.4880 30180279

[39] Boswell JF, Thompson-Hollands J, Farchione TJ, Barlow DH. Intolerance of uncertainty: a common factor in the treatment of emotional disorders. J Clin Psychol. 2013;69(6):630-645. doi:10.1002/jclp.21965 23381685 PMC3712497

[40] Buhr K, Dugas MJ. Investigating the construct validity of intolerance of uncertainty and its unique relationship with worry. J Anxiety Disord. 2006;20(2):222-236. doi:10.1016/j.janxdis.2004.12.004 16464706

[41] Carleton RN, Collimore KC, Asmundson GJG. “It’s not just the judgements—it’s that I don’t know”: intolerance of uncertainty as a predictor of social anxiety. J Anxiety Disord. 2010;24(2):189-195. doi:10.1016/j.janxdis.2009.10.007 19931391

[42] Carleton RN. The intolerance of uncertainty construct in the context of anxiety disorders: theoretical and practical perspectives. Expert Rev Neurother. 2012;12(8):937-947. doi:10.1586/ern.12.82 23002938

[43] Carleton RN, Mulvogue MK, Thibodeau MA, McCabe RE, Antony MM, Asmundson GJG. Increasingly certain about uncertainty: intolerance of uncertainty across anxiety and depression. J Anxiety Disord. 2012;26(3):468-479. doi:10.1016/j.janxdis.2012.01.011 22366534

[44] Carleton RN. Into the unknown: a review and synthesis of contemporary models involving uncertainty. J Anxiety Disord. 2016;39:30-43. doi:10.1016/j.janxdis.2016.02.007 26945765

[45] Milne S, Lomax C, Freeston MH. A review of the relationship between intolerance of uncertainty and threat appraisal in anxiety. Cognitive Behavior Therapist. 2019;12:e38. doi:10.1017/S1754470X19000230

[46] Pepperdine E, Lomax C, Freeston MH. Disentangling intolerance of uncertainty and threat appraisal in everyday situations. J Anxiety Disord. 2018;57:31-38. doi:10.1016/j.janxdis.2018.04.002 29724665

[47] Chen JTH, Lovibond PF. Intolerance of uncertainty is associated with increased threat appraisal and negative affect under ambiguity but not uncertainty. Behav Ther. 2016;47(1):42-53. doi:10.1016/j.beth.2015.09.004 26763496

[48] Bottesi G, Ghisi M, Carraro E, Barclay N, Payne R, Freeston MH. Revising the intolerance of uncertainty model of generalized anxiety disorder: evidence from UK and Italian undergraduate samples. Front Psychol. 2016;7:1723. doi:10.3389/fpsyg.2016.01723 27847496 PMC5088195

[49] Phillips SM, Padgett LS, Leisenring WM, . Survivors of childhood cancer in the United States: prevalence and burden of morbidity. Cancer Epidemiol Biomarkers Prev. 2015;24(4):653-663. doi:10.1158/1055-9965.EPI-14-1418 25834148 PMC4418452

[50] National Cancer Institute. Common Terminology Criteria for Adverse Events v4.0. Published online 2009. Accessed March 18, 2024. https://ctep.cancer.gov/protocoldevelopment/electronic_applications/ctc.htm#ctc_40

[51] Simard S, Savard J. Screening and comorbidity of clinical levels of fear of cancer recurrence. J Cancer Surviv. 2015;9(3):481-491. doi:10.1007/s11764-015-0424-4 25603948

[52] Fardell JE, Jones G, Smith AB, . Exploring the screening capacity of the Fear of Cancer Recurrence Inventory-Short Form for clinical levels of fear of cancer recurrence. Psychooncology. 2018;27(2):492-499. doi:10.1002/pon.4516 28755462

[53] Treede RD, Rief W, Barke A, . A classification of chronic pain for *ICD-11*. Pain. 2015;156(6):1003-1007. doi:10.1097/j.pain.0000000000000160 25844555 PMC4450869

[54] Steingrímsdóttir ÓA, Landmark T, Macfarlane GJ, Nielsen CS. Defining chronic pain in epidemiological studies: a systematic review and meta-analysis. Pain. 2017;158(11):2092-2107. doi:10.1097/j.pain.0000000000001009 28767506

[55] Kroenke K, Spitzer RL. The PHQ-9: a new depression diagnostic and severity measure. Psychiatr Ann. 2002;32(9):509-515. doi:10.3928/0048-5713-20020901-06

[56] Kroenke K, Spitzer RL, Williams JB. The PHQ-9: validity of a brief depression severity measure. J Gen Intern Med. 2001;16(9):606-613. doi:10.1046/j.1525-1497.2001.016009606.x 11556941 PMC1495268

[57] Kroenke K, Strine TW, Spitzer RL, Williams JBW, Berry JT, Mokdad AH. The PHQ-8 as a measure of current depression in the general population. J Affect Disord. 2009;114(1-3):163-173. doi:10.1016/j.jad.2008.06.026 18752852

[58] Spitzer RL, Kroenke K, Williams JB, Lowe B. A brief measure for assessing generalized anxiety disorder: the GAD-7. Arch Intern Med. 2006;166(10):1092-1097. doi:10.1001/archinte.166.10.1092 16717171

[59] Ware JE, Snow KK, Kosinski M, Gandek B. SF-36 Health Survey: Manual and Interpretation Guide. Quality Metric Inc; 2003.

[60] Carleton RN, Norton MAPJ, Asmundson GJG. Fearing the unknown: a short version of the Intolerance of Uncertainty Scale. J Anxiety Disord. 2007;21(1):105-117. doi:10.1016/j.janxdis.2006.03.014 16647833

[61] Wilson EJ, Stapinski L, Dueber DM, Rapee RM, Burton AL, Abbott MJ. Psychometric properties of the Intolerance of Uncertainty Scale-12 in generalized anxiety disorder: assessment of factor structure, measurement properties and clinical utility. J Anxiety Disord. 2020;76:102309. doi:10.1016/j.janxdis.2020.102309 33002756

[62] Buysse DJ, Yu L, Moul DE, . Development and validation of patient-reported outcome measures for sleep disturbance and sleep-related impairments. Sleep. 2010;33(6):781-792. doi:10.1093/sleep/33.6.781 20550019 PMC2880437

[63] Yu L, Buysse DJ, Germain A, . Development of short forms from the PROMISTM Sleep Disturbance and Sleep-Related Impairment item banks. Behav Sleep Med. 2012;10(1):6-24. doi:10.1080/15402002.2012.636266 PMC3261577 22250775

[64] Zou G. A modified Poisson regression approach to prospective studies with binary data. Am J Epidemiol. 2004;159(7):702-706. doi:10.1093/aje/kwh090 15033648

[65] Hayes AF. Introduction to Mediation, Moderation, and Conditional Process Analysis: A Regression-Based Approach. 3rd ed. The Guilford Press; 2022.

[66] Benjamini Y, Hochberg Y. Controlling the false discovery rate: a practical and powerful approach to multiple testing. J R Stat Soc Series B Stat Methodol. 1995;57(1):289-300. doi:10.1111/j.2517-6161.1995.tb02031.x

[67] Rudy L, Maheu C, Körner A, Lebel S, Gélinas C. The FCR-1: initial validation of a single-item measure of fear of cancer recurrence. Psychooncology. 2020;29(4):788-795. doi:10.1002/pon.5350 32026563

[68] Smith A, Gao M, Tran M, . Evaluation of the validity and screening performance of a revised single-item fear of cancer recurrence screening measure (FCR-1r). Psycho-Oncology. 2023;32(6):961-971. doi:10.1002/pon.6139 37120796

[69] Borsook D. Neurological diseases and pain. Brain. 2012;135(2):320-344. doi:10.1093/brain/awr271 22067541 PMC3281476

[70] Snyder H, Robinson K, Shah D, Brennan R, Handrigan M. Signs and symptoms of patients with brain tumors presenting to the emergency department. J Emerg Med. 1993;11(3):253-258. doi:10.1016/0736-4679(93)90042-6 8340578

[71] Alther B, Mylius V, Weller M, Gantenbein A. From first symptoms to diagnosis: initial clinical presentation of primary brain tumors. Clinical and Translational Neuroscience. 2020;4(2):2514183X2096836. doi:10.1177/2514183X20968368

[72] Bye A, Tropé C, Jon HL. Health-related quality of life and occurrence of intestinal side effects after pelvic radiotherapy: evaluation of long-term effects of diagnosis and treatment. Acta Oncol (Madr). 2000;39(2):173-180. doi:10.1080/028418600430734 10859007

[73] Gami B, Harrington K, Blake P, . How patients manage gastrointestinal symptoms after pelvic radiotherapy. Aliment Pharmacol Ther. 2003;18(10):987-994. doi:10.1046/j.1365-2036.2003.01760.x 14616164

[74] Lobo C, Pinto J, Morcerf B, . Pulmonary hypertension in sickle cell disease: study of 137 patients randomly selected from a public hematology hospital in Rio de Janeiro, Brazil. Haematologica. 2011;96:625.

[75] Astin M, Griffin T, Neal RD, Rose P, Hamilton W. The diagnostic value of symptoms for colorectal cancer in primary care: a systematic review. Br J Gen Pract. 2011;61(586):e231-e243. doi:10.3399/bjgp11X572427 21619747 PMC3080228

[76] Yaxley J. Urinary tract cancers: an overview for general practice. J Family Med Prim Care. 2016;5(3):533-538. doi:10.4103/2249-4863.197258 28217578 PMC5290755

[77] Penninx BW, Pine DS, Holmes EA, Reif A. Anxiety disorders. Lancet. 2021;397(10277):914-927. doi:10.1016/S0140-6736(21)00359-7 33581801 PMC9248771

[78] Tauber NM, O’Toole MS, Dinkel A, . Effect of psychological intervention on fear of cancer recurrence: a systematic review and meta-analysis. J Clin Oncol. 2019;37(31):2899-2915. doi:10.1200/JCO.19.00572 31532725 PMC6823887

[79] Paperák P, Javůrková A, Raudenská J. Therapeutic intervention in fear of cancer recurrence in adult oncology patients: a systematic review. J Cancer Surviv. 2022;17:1017-1035. doi:10.1007/s11764-022-01277-x 36307611

[80] Hall DL, Luberto CM, Philpotts LL, Song R, Park ER, Yeh GY. Mind-body interventions for fear of cancer recurrence: a systematic review and meta-analysis. Psychooncology. 2018;27(11):2546-2558. doi:10.1002/pon.4757 29744965 PMC6488231

[81] Lichtenthal WG, Corner GW, Slivjak ET, . A pilot randomized controlled trial of cognitive bias modification to reduce fear of breast cancer recurrence. Cancer. 2017;123(8):1424-1433. doi:10.1002/cncr.30478 28055119 PMC5391320

[82] O’Laughlin KD, Martin MJ, Ferrer E. Cross-sectional analysis of longitudinal mediation processes. Multivariate Behav Res. 2018;53(3):375-402. doi:10.1080/00273171.2018.1454822 29624079

