# Supplementary material for: Fear of Cancer Recurrence in Adult Survivors of Childhood Cancer
Source: JAMA Netw Open. 2024 Oct 3;7(10):e2436144. doi: 10.1001/jamanetworkopen.2024.36144 (PMC11450519; doi:10.1001/jamanetworkopen.2024.36144)
Supplement: Supplement 1. — eAppendix 1. Additional Information Regarding Selection of Demographic Variables eAppendix 2. Additional Information About Psychometric Properties and Prior Use of Study Measures eTable 1. Items on the Fear of Cancer Recurrence Inventory - Short Form eTable 2. Demographics and Clinical Characteristics of Participants and Non-Participants eTable 3. Univariate Models Assessing Risk Factors for Clinically Significant Fear of Cancer Recurrence eTable 4. Models for Intolerance of Uncertainty as a Mediator of the Effect of Elevated Anxiety and/or Depression on Clinical Fear of Cancer Recurrence eFigure. Participant Flow Diagram eReferences [file jamanetwopen-e2436144-s001.pdf]

Supplemental Online Content

Pizzo A, Leisenring WM, Stratton KL, et al. Fear of cancer recurrence in adult survivors of childhood cancer. *JAMA Netw Open.* 2024;7(9):e2436144.
doi:10.1001/jamanetworkopen.2024.36144

eAppendix 1. Additional Information Regarding Selection of Demographic Variables

eAppendix 2. Additional Information About Psychometric Properties and Prior Use of Study Measures

eTable 1. Items on the Fear of Cancer Recurrence Inventory - Short Form

eTable 2. Demographics and Clinical Characteristics of Participants and Non-Participants

eTable 3. Univariate Models Assessing Risk Factors for Clinically Significant Fear of Cancer Recurrence

eTable 4. Models for Intolerance of Uncertainty as a Mediator of the Effect of Elevated Anxiety and/or Depression on Clinical Fear of Cancer Recurrence

eFigure. Participant flow diagram

eReferences

This supplemental material has been provided by the authors to give readers additional information about their work.

© 2024 Pizzo A et al. *JAMA Network Open.*

eAppendix 1

Additional Information Regarding Selection of Demographic Variables

Given the lack of research on fear of cancer recurrence in childhood cancer survivors, selection of relevant demographic variables was based on prior studies conducted among survivors of adult-onset cancer.[1,2] Demographic variables chosen had shown to be significantly associated with fear of cancer recurrence in prior studies among survivors of adult-onset cancer.

© 2024 Pizzo A et al. *JAMA Network Open.*

eAppendix 2

Additional Information About Psychometric Properties and Prior Use of Study Measures

Fear of Cancer Recurrence Inventory - Short Form
The FCRI-SF has excellent internal consistency, convergent validity, and divergent validity.[3–5] Internal consistency was high in the current study (α=0.92). The FCRI-SF is one of the most commonly used fear of cancer recurrence measures in adult-onset cancer and has been used in multiple prior studies of fear of cancer recurrence, including with survivors of breast, lung, colorectal, pancreatic, prostate, head and neck, and melanoma cancer.[5]

Patient Health Questionnaire-8
The PHQ-8 has excellent psychometric properties, including excellent internal reliability, construct validity, and criterion validity.[6,7] Internal consistency was high within the study sample (α=0.90). The PHQ-8 has been previously used to measure depressive symptoms in **adolescent and young adult survivors of cancer and survivors of adult-onset cancer.**[8–11]

Generalized Anxiety Disorder-7
The Generalized Anxiety Disorder 7-item (GAD-7) has good psychometric properties including excellent convergent validity and internal consistency.[12] Internal consistency was high within the study sample (α=0.93). Prior studies have used the GAD-7 to measure anxiety among childhood and adult-onset cancer survivors (Baclig et al., 2023; Burghardt et al., 2019; Esser et al., 2017; Götze et al., 2019; Kuba et al., 2019; Yusof et al., 2023).[8,10,11,13–15]

Intolerance of Uncertainty Scale-12
The Intolerance of Uncertainty Scale 12-item (IUS-12) has been shown to have excellent internal consistency and convergent validity.[16,17] Internal consistency was high (α=0.93). The IUS-12 has also been used in prior studies of childhood and adult-onset cancer survivors.[18,19]

PROMIS Sleep Disturbance - Short Form
The National Institute of Health Patient Reported Outcomes Measurement Information System – Sleep Disturbance (PROMIS-SD) – Short Form 8-item has strong internal consistency and convergent validity.[20,21] Internal consistency was high (α=0.88). It has also been used to measure sleep disturbance in previous studies with adult-onset cancer survivors.[22–24]

Chronic Pain
Chronic pain was assessed via two items (i.e., "Do you have any persistent or recurrent pain, more than aches and pains that are fleeting?" and "How long have you been experiencing pain?").[25,26] These items are based on the definition of chronic pain from the International Association of Pain and are recommended for use in epidemiological studies of chronic pain.[25,26]

eTable 1

**Items on the Fear of Cancer Recurrence Inventory - Short Form**
1. I am worried or anxious about the possibility of cancer recurrence.
2. I am afraid of cancer recurrence.
3. I believe it is normal to be worried or anxious about the possibility of cancer recurrence.
4. When I think about the possibility of cancer recurrence, this triggers other unpleasant thoughts or images (such as death, suffering, the consequences for my family).
5. I believe that I am cured and that the cancer will not come back.
6. In your opinion, are you at risk of having a cancer recurrence?
7. How often do you think about the possibility of cancer recurrence?
8. How much time per day do you spend thinking about the possibility of cancer recurrence?
9. How long have you been thinking about the possibility of cancer recurrence?

Note. Instructions provided to participants = "*Most people who have been diagnosed with cancer are worried, to varying degrees, that there might be a recurrence of the cancer. By recurrence, we mean the possibility that the cancer could return or progress in the same place or in another part of the body. This questionnaire aims to better understand the experience of worries about cancer recurrence. Please read each statement and indicate to what degree it applied to you DURING THE PAST MONTH by circling the appropriate number*". Item 5 is reverse coded.

© 2024 Pizzo A et al. *JAMA Network Open.*

eTable 2

Demographics and Clinical Characteristics of Participants and Non-Participants

	Participants (n=229)		Non-Participants (n=466)	
	**n**	**%**	**n**	**%**
**Sex**				
Male	114	49.8	248	53.2
Female	115	50.2	218	46.8
**Race and Ethnicity***				
American Indian	1	0.4	2	0.4
Asian Indian	0	0.0	1	0.2
Asian or Pacific Islander, Type Unknown	0	0.0	4	0.9
Black	6	2.6	29	6.2
Chinese	1	0.4	4	0.9
Hispanic: Mexican, Mexican American, Chicano	3	1.3	11	2.4
Hispanic: Cuban	1	0.4	0	0.0
Hispanic: Puerto Rican	0	0.0	3	0.6
Hispanic: Another Hispanic, Latino or Spanish Origin	5	2.2	15	3.2
Hispanic: type unknown	2	0.9	14	3.0
Vietnamese	0	0.0	1	0.2
White, non-Hispanic	205	89.5	369	79.2
White, Hispanic Ethnicity Unknown	5	2.2	13	2.8
**Education**				
Completed high school	29	12.7	76	16.3
Some college/college graduate	148	64.6	299	64.3
Post-graduate	52	22.7	90	19.4
**Employment**				
Full-Time	139	66.5	221	62.1
Part-Time	30	14.4	58	16.2
Not Employed	40	19.1	77	21.6
**Diagnosis**				
Leukemia	79	34.5	139	29.8
CNS Tumor	24	10.5	65	13.9
Lymphomas (HD, NHL)	47	20.5	99	21.2
Wilms, Neuroblastoma, STS	51	22.3	121	26.0
Bone Cancer	28	12.2	42	9.0
**Married***				
Yes	129	63.2	189	54.0
No	75	36.8	161	46.0
**Location**				
Metropolitan (RUCA 1-3)	177	79.7	348	81.1
Nonmetropolitan (RUCA 4-10)	45	20.3	81	18.9
**Endocrine Condition (grade 2-4)***				
Yes	80	34.9	124	26.6
No	149	65.1	342	73.4
**Respiratory Condition (grade 2-4)**				
Yes	22	9.6	37	7.9

© 2024 Pizzo A et al. *JAMA Network Open.*

	Participants (n=229)		Non-Participants (n=466)	
	**n**	**%**	**n**	**%**
No	207	90.4	429	92.1
**Cardiovascular Condition (grade 2-4)**				
Yes	77	33.6	139	29.8
No	152	66.4	327	70.2
**GI Condition (grade 2-4)**				
Yes	27	11.8	37	7.9
No	202	88.2	429	92.1
**Musculoskeletal Condition (grade 2-4)**				
Yes	20	8.7	27	5.8
No	209	91.3	439	94.2
**Neurological Condition (grade 2-4)**				
Yes	34	14.8	71	15.2
No	195	85.2	395	84.8
**Physical Health Status**				
Poor, Fair	34	15.1	58	12.6
Good, Very Good, Excellent	191	84.9	401	87.4
**Chemotherapy**				
Yes	186	85.3	356	82.0
No	32	14.7	78	18.0
**Vinca alkaloids**				
Yes	162	74.3	296	68.8
No	56	25.7	134	31.2
**Platinum**				
Yes	26	11.9	53	12.2
No	192	88.1	383	87.8
**IV Methotrexate ≥ 10000mg/m^2**				
Yes	25	11.7	41	9.6
No	189	88.3	385	90.4
**IT Methotrexate**				
Yes	89	41.0	158	36.4
No	128	59.0	276	63.6
**Radiation**				
Yes	98	44.7	215	49.3
No	121	55.3	221	50.7
**Cranial Radiation**				
Yes	46	21.1	115	26.9
No	172	78.9	312	73.1
**Other Radiation to Head**				
Yes	10	4.6	30	7.0
No	208	95.4	397	93.0
**Neck Radiation**				
Yes	32	14.7	74	17.3
No	186	85.3	353	82.7
**Chest Radiation**				
Yes	34	15.6	70	16.4
No	184	84.4	357	83.6
**Abdomen Radiation**				
Yes	35	16.1	82	19.2
No	183	83.9	345	80.8
**Pelvis Radiation**				

© 2024 Pizzo A et al. *JAMA Network Open.*

	Participants (n=229)		Non-Participants (n=466)	
	**n**	**%**	**n**	**%**
Yes	25	11.5	64	15.0
No	193	88.5	363	85.0
**Limb Radiation**				
Yes	8	3.7	16	3.7
No	210	96.3	411	96.3
**Non-Brain Radiation**				
Yes	63	28.9	130	30.4
No	155	71.1	297	69.6
**Surgery**				
Yes	162	70.7	320	74.1
No	67	29.3	112	25.9
**Amputation**				
Yes	12	5.2	12	2.6
No	217	94.8	454	97.4
**Limb Sparing**				
Yes	11	4.8	21	4.5
No	218	95.2	445	95.5
**Any Recurrence of Original Cancer**				
Yes	21	9.2	28	6.0
No	208	90.8	438	94.0
**Any SMN (non-NMSC)**				
Yes	17	7.4	28	6.0
No	212	92.6	438	94.0

Note. Non-participants were members of the Childhood Cancer Survivorship who were invited to participate in the current study but did not consent or complete study procedures; HL = Hodgkin Lymphoma; NHL = Non-Hodgkin Lymphoma; STS = Sarcomas, Soft Tissue; SMN = Second Malignant Neoplasms; NMSC= Non-Melanoma Skin Cancer; Percentages among those with known values; * = $p < 0.05$ based on chi-square or Fisher's exact comparison, as appropriate.

© 2024 Pizzo A et al. *JAMA Network Open.*

eTable 3

Univariate Models Assessing Risk Factors for Clinically Significant Fear of Cancer Recurrence

Risk Factor	Prevalence Ratio	95% CI	p-value
**Sex**			
Female	1.9	1.0, 3.7	0.04
Male	1.0	-	-
**Race**			
Other	0.7	0.2, 2.2	0.53
White, non-Hispanic	1.0	-	-
**Education**			
Completed high school	7.0	1.5, 32.8	0.01
Some college/college graduate	4.8	1.2, 19.5	0.03
Post-graduate	1.0	-	-
**Diagnosis**			
Leukemia	0.8	0.3, 2.0	0.60
CNS	1.6	0.6, 4.1	0.33
Lymphomas (HD, NHL)	0.8	0.3, 2.2	0.68
Bone cancer	1.6	0.6, 3.9	0.31
Wilms, neuroblastoma, STS	1.0	-	-
**Metro or non-metro (RUCA)**			
Nonmetropolitan (RUCA 4-10)	2.2	1.1, 4.4	0.028
Metropolitan (RUCA 1-3)	1.0	-	-
**Chronic Pain**			
Yes	2.9	1.5, 5.5	<0.01
No	1.0	-	-
**Depression**			
Mild	3.0	1.4, 6.2	<0.01
Elevated	5.1	2.6, 10.2	<0.01
Minimal	1.0	-	-
**Anxiety**			
Mild	3.1	1.4, 6.8	<0.01
Elevated	6.9	3.8, 12.7	<0.01
Minimal	1.0	-	-
**Depression and Anxiety**			
Either Elevated	3.8	1.9, 7.6	<0.01
Both Elevated	7.5	3.8, 14.6	<0.01
Neither Elevate	1.0	-	-
**Employment Status**			
Employed Part-Time	0.7	0.2, 2.4	0.61
Not Employed	2.4	1.2, 4.6	0.01
Employed Full-Time	1.0	-	-
**Married**			
Yes	1.0	0.5, 2.1	0.90
No	1.0	-	-
**Any Chronic Condition (grade 2-4)**			
Yes	1.3	0.6, 3.1	0.50
No	1.0	-	-
**Endocrine Condition (grade 2-4)**			
Yes	1.3	0.7, 2.4	0.48
No	1.0	-	-
**Respiratory Condition (grade 2-4)**			
Yes	1.4	0.6, 3.4	0.46
No	1.0	-	-
**Cardiovascular Condition**			

© 2024 Pizzo A et al. *JAMA Network Open.*

Risk Factor	Prevalence Ratio	95% CI	p-value
Yes	1.1	0.6, 2.2	0.68
No	1.0	-	-
**GI Condition (grade 2-4)**			
Yes	1.2	0.5, 2.9	0.66
No	1.0	-	-
**Musculoskeletal Condition (grade 2-4)**			
Yes	0.9	0.3, 2.8	0.90
No	1.0	-	-
**Neurological Condition (grade 2-4)**			
Yes	3.5	1.9, 6.5	<0.01
No	1.0	-	-
**Health Status**			
Fair, Poor	5.6	3.2, 9.8	<0.01
Excellent, Very Good, Good	1.0	-	-
**Chemotherapy**			
Yes	0.9	0.4, 1.9	0.71
No	1.0	-	-
**Vinca Alkaloids**			
Yes	0.5	0.3, 1.0	0.05
No	1.0	-	-
**Platinum**			
Yes	1.6	0.7, 3.5	0.26
No	1.0	-	-
**IV Methotrexate >10000 mg/m2**			
Yes	0.5	0.1, 1.6	0.23
No	1.0	-	-
**IT Methotrexate**			
Yes	0.8	0.4, 1.5	0.44
No	1.0	-	-
**Radiation**			
Yes	2.0	1.1, 3.8	1.97
No	1.0	-	-
**Cranial Radiation**			
Yes	0.9	0.4, 1.9	0.70
No	1.0	-	-
**Other Head Radiation**			
Yes	0.5	0.1, 3.5	0.48
No	1.0	-	-
**Neck Radiation**			
Yes	1.2	0.5, 2.7	0.71
No	1.0	-	-
**Chest Radiation**			
Yes	1.1	0.5, 2.5	0.82
No	1.0	-	-
**Abdomen Radiation**			
Yes	1.5	0.7, 3.1	0.28
No	1.0	-	-
**Pelvis Radiation**			
Yes	2.5	1.2, 4.9	0.01
No	1.0	-	-
**Limb Radiation**			
Yes	1.2	0.3, 4.9	0.80
No	1.0	-	-
**Non-Brain Radiation**			

© 2024 Pizzo A et al. *JAMA Network Open.*

Risk Factor	Prevalence Ratio	95% CI	p-value
Yes	1.4	0.8, 2.8	0.27
No	1.0	-	-
**Surgery**			
Yes	1.0	0.4, 2.2	0.95
No	1.0	-	-
**Amputation or Limp Sparing**			
Yes	2.1	1.1, 4.4	0.04
No	1.0	-	-
**Other Major Surgery**			
Yes	1.4	0.7, 2.8	0.39
No	1.0	-	-
**Intolerance of Uncertainity**	1.1	1.0, 1.1	<0.01
**Age at cancer diagnosis**	1.0	1.0, 1.1	0.55
**Age at EASE questionnaire**	1.0	1.0, 1.0	0.96
**Time since cancer diagnosis (years)**	1.0	1.0, 1.0	0.59
**Sleep T-score(PROMIS-SD)**	1.1	1.0, 1.2	0.19
**Age at cancer diagnosis (categories)**			
13-20	1.1	0.5, 2.2	0.84
5-12	0.7	0.3, 1.5	0.32
0-4	1.0	-	-
**Any Recurrence**			
Yes	1.4	0.6, 3.4	0.48
No	1.0	-	-
**Any SMN (non-NMSC)**			
Yes	0.7	0.2, 2.8	0.66
No	1.0	-	-
**Any Recurrence or SMN**			
Yes	1.2	0.6, 2.6	0.62
No	1.0	-	-

Note. CNS = Central Nervous System; HL = Hodgkin Lymphoma; NHL = Non-Hodgkin Lymphoma; STS = Sarcomas, Soft Tissue; SMN = Second Malignant Neoplasms.

© 2024 Pizzo A et al. *JAMA Network Open.*

eTable 4

Models for Intolerance of Uncertainty as a Mediator of the Effect of Elevated Anxiety and/or Depression on Clinical Fear of Cancer Recurrence

		Direct Effect (c′)		Total Effect (c)		Indirect Effect (a*b)	
**Model**	**Exposure**	**β (95% CI)**	**p-value**	**β (95% CI)**	**p-value**	**Est (95%CI)**	**p-value**
**A**							
	Either Anxiety & Depression (Elevated) vs. neither	0.98 (0.13, 1.83)	0.023	1.25 (0.46, 2.04)	0.002	0.23 (-0.06, 0.52)	0.13
	Both Anxiety & Depression (Moderate/Severe) vs. neither	1.55 (0.71, 2.38)	<0.001	2.00 (1.33, 2.66)	<0.001	0.40 (-0.05, 0.86)	0.08
**B**							
	Depression Only (Elevated) vs. neither	0.99 (-0.03, 2.01)	0.06	1.13 (0.12, 2.11)	0.03	0.10 (-0.08, 0.28)	0.28
	Anxiety Only (Elevated) vs. neither	0.97 (0.01, 1.96)	0.05	1.38 (0.45, 2.31)	0.004	0.39 (-0.04, 0.82)	0.08
	Both Anxiety & Depression (Elevated) vs. neither	1.54 (0.71, 2.38)	<0.001	2.00 (1.33, 2.66)	<0.001	0.41 (-0.02, 0.84)	0.06
**C**							
	Depression Only (Elevated) vs. neither	0.98 (-0.03, 2.00)	0.06	1.12 (0.12, 2.11)	0.03	0.11 (-0.08, 0.29)	0.26
	Anxiety Only or Both Anxiety & Depression (Elevated) vs. neither	1.37 (0.57, 2.17)	<0.001	1.83 (1.17, 2.48)	<0.001	0.42 (0.02, 0.82)	0.04

Note. Model C presented in the manuscript; β = Beta; Est = Estimate; p-value and confidence interval for indirect effect based on 1000 bootstraps.

© 2024 Pizzo A et al. *JAMA Network Open.*

eFigure

Participant flow diagram

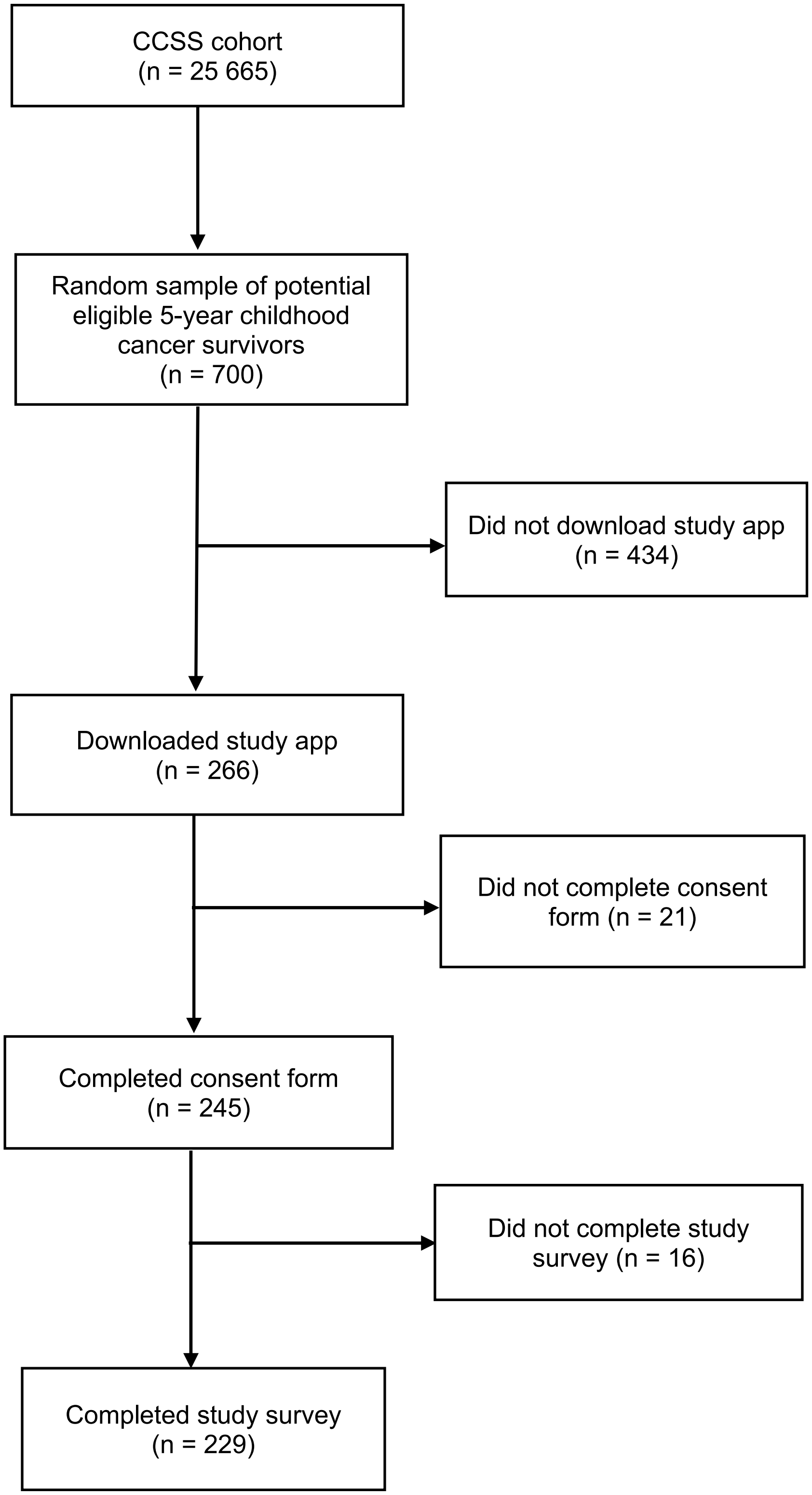

© 2024 Pizzo A et al. *JAMA Network Open.*

eReferences

1. Simard S, Thewes B, Humphris G, et al. Fear of cancer recurrence in adult cancer survivors: a systematic review of quantitative studies. *J Cancer Surviv*. 2013;7(3):300-322. doi:10.1007/s11764-013-0272-z

2. Koch L, Jansen L, Brenner H, Arndt V. Fear of recurrence and disease progression in long-term (≥5 years) cancer survivors-a systematic review of quantitative studies: Fear of recurrence and disease progression in long-term cancer survivors. *Psycho-Oncology*. 2013;22(1):1-11. doi:10.1002/pon.3022

3. Fardell JE, Jones G, Smith AB, et al. Exploring the screening capacity of the Fear of Cancer Recurrence Inventory-Short Form for clinical levels of fear of cancer recurrence. *Psycho-Oncology*. 2018;27(2):492-499. doi:10.1002/pon.4516

4. Simard S, Savard J. Screening and comorbidity of clinical levels of fear of cancer recurrence. *J Cancer Surviv*. 2015;9(3):481-491. doi:10.1007/s11764-015-0424-4

5. Smith AB, Costa D, Galica J, et al. Spotlight on the Fear of Cancer Recurrence Inventory (FCRI). *PRBM*. 2020;Volume 13:1257-1268. doi:10.2147/PRBM.S231577

6. Kroenke K, Spitzer RL, Williams JB. The PHQ-9: validity of a brief depression severity measure. *Journal of general internal medicine*. 2001;16(9):606-613.

7. Kroenke K, Strine TW, Spitzer RL, Williams JBW, Berry JT, Mokdad AH. The PHQ-8 as a measure of current depression in the general population. *Journal of Affective Disorders*. 2009;114(1-3):163-173. doi:10.1016/j.jad.2008.06.026

8. Baclig NV, Comulada WS, Ganz PA. Mental health and care utilization in survivors of adolescent and young adult cancer. *JNCI Cancer Spectrum*. 2023;7(6):pkad098. doi:10.1093/jncics/pkad098

9. Cheruvu VK, Oancea SC. Current depression as a potential barrier to health care utilization in adult cancer survivors. *Cancer Epidemiology*. 2016;44:132-137. doi:10.1016/j.canep.2016.08.012

10. Kuba K, Esser P, Mehnert A, et al. Risk for depression and anxiety in long-term survivors of hematologic cancer. *Health Psychology*. 2019;38(3):187-195. doi:10.1037/hea0000713

11. Yusof KM, Mohd Sidik S, Mahmud R, Abdullah M, Avery-Kiejda KA, Rosli R. Association of psychological distress with arm morbidity symptoms in breast cancer survivors: outcomes from the use of PHQ-9 and GAD-7 questionnaires. *Breast Cancer*. 2023;30(5):810-819. doi:10.1007/s12282-023-01475-0

12. Spitzer RL, Kroenke K, Williams JB, Lowe B. A brief measure for assessing generalized anxiety disorder: the GAD-7. *Archives of internal medicine*. 2006;166(10):1092-1097. doi:10.1001/archinte.166.10.1092

13. Burghardt J, Klein E, Brähler E, et al. Prevalence of mental distress among adult survivors of childhood cancer in Germany—Compared to the general population. *Cancer Medicine*. 2019;8(4):1865-1874. doi:10.1002/cam4.1936

14. Esser P, Hartung TJ, Friedrich M, et al. The GENERALIZED ANXIETY DISORDER SCREENER (GAD-7) and the anxiety module of the HOSPITAL AND DEPRESSION SCALE (HADS-A) as screening tools for generalized anxiety disorder among cancer patients. *Psycho-Oncology*. 2018;27(6):1509-1516. doi:10.1002/pon.4681

15. Götze H, Taubenheim S, Dietz A, Lordick F, Mehnert-Theuerkauf A. Fear of cancer recurrence across the survivorship trajectory: Results from a survey of adult long-term cancer survivors. *Psycho-Oncology*. 2019;28(10):2033-2041. doi:10.1002/pon.5188

© 2024 Pizzo A et al. *JAMA Network Open.*

16. Carleton RN, Norton MAPJ, Asmundson GJG. Fearing the unknown: A short version of the Intolerance of Uncertainty Scale. *Journal of Anxiety Disorders*. 2007;21(1):105-117. doi:10.1016/j.janxdis.2006.03.014

17. Wilson EJ, Stapinski L, Dueber DM, Rapee RM, Burton AL, Abbott MJ. Psychometric properties of the Intolerance of Uncertainty Scale-12 in generalized anxiety disorder: Assessment of factor structure, measurement properties and clinical utility. *Journal of Anxiety Disorders*. 2020;76:102309. doi:10.1016/j.janxdis.2020.102309

18. Lebel S, Maheu C, Tomei C, et al. Towards the validation of a new, blended theoretical model of fear of cancer recurrence. *Psycho-Oncology*. 2018;27(11):2594-2601. doi:10.1002/pon.4880

19. Tutelman PR, Chambers CT, Heathcote LC, et al. Measuring fear of cancer recurrence in survivors of childhood cancer: Development and preliminary validation of the Fear of Cancer Recurrence Inventory (FCRI)-Child and Parent versions. *Psycho-Oncology*. 2022;31(6):911-919. doi:10.1002/pon.5879

20. Buysse DJ, Yu L, Moul DE, et al. Development and validation of patient-reported outcome measures for sleep disturbance and sleep-related impairments. *Sleep*. 2010;33(6):781-792. doi:10.1093/sleep/33.6.781

21. Yu L, Buysse DJ, Germain A, et al. Development of Short Forms From the PROMIS™ Sleep Disturbance and Sleep-Related Impairment Item Banks. *Behavioral Sleep Medicine*. 2012;10(1):6-24. doi:10.1080/15402002.2012.636266

22. Bock K, Peltzer J, Liu W, Colgrove Y, Smirnova I, Siengsukon C. Sleep quality and lymphedema in breast cancer survivors: a mixed method analysis. *J Cancer Surviv*. Published online January 6, 2024. doi:10.1007/s11764-023-01516-9

23. Coles T, Bennett AV, Tan X, et al. Relationship between sleep and exercise as colorectal cancer survivors transition off treatment. *Support Care Cancer*. 2018;26(8):2663-2673. doi:10.1007/s00520-018-4110-8

24. Schreier AM, Johnson LA, Vohra NA, Muzaffar M, Kyle B. Post-Treatment Symptoms of Pain, Anxiety, Sleep Disturbance, and Fatigue in Breast Cancer Survivors. *Pain Management Nursing*. 2019;20(2):146-151. doi:10.1016/j.pmn.2018.09.005

25. Treede RD, Rief W, Barke A, et al. A classification of chronic pain for ICD-11. *Pain*. 2015;156(6):1003-1007. doi:10.1097/j.pain.0000000000000160

26. Steingrímsdóttir ÓA, Landmark T, Macfarlane GJ, Nielsen CS. Defining chronic pain in epidemiological studies: a systematic review and meta-analysis. *Pain*. 2017;158(11):2092-2107. doi:10.1097/j.pain.0000000000001009

© 2024 Pizzo A et al. *JAMA Network Open.*
